# Terahertz and Raman Spectroscopic Investigation of Monohydrate Cocrystal of Antitubercular Isoniazid with Protocatechuic Acid

**DOI:** 10.3390/pharmaceutics13081303

**Published:** 2021-08-20

**Authors:** Yanhua Bo, Jiyuan Fang, Ziming Zhang, Jiadan Xue, Jianjun Liu, Zhi Hong, Yong Du

**Affiliations:** 1 Centre for THz Research, China Jiliang University, Hangzhou 310018, China; bobo6687@163.com (Y.B.); jiyuanfang@foxmail.com (J.F.); ziming_zhang@yeah.net (Z.Z.); jianjun@cjlu.edu.cn (J.L.); hongzhi@cjlu.edu.cn (Z.H.); 2 Department of Chemistry, Zhejiang Sci-Tech University, Hangzhou 310018, China; jenniexue@126.com

**Keywords:** isoniazid (INH), protocatechuic acid (PA), monohydrate cocrystal, THz time-domain spectroscopy (THz-TDS), Raman spectroscopy, density functional theory (DFT)

## Abstract

Pharmaceutical cocrystal provides an alternative modification strategy for the formulation development of drugs owning to their potential ability to improve the physicochemical properties of active pharmaceutical ingredients (APIs) efficiently by changing inter-molecular interactions between raw materials. Isoniazid (INH) is an indispensable main drug for the treatment of tuberculosis, but its tablet formulation is unstable and prone to degradation. In the present study, the monohydrate cocrystal of INH and protocatechuic acid (PA) was prepared by solvent evaporation using PA as cocrystal former to optimize the properties of INH. The parent materials and corresponding 1:1 molar ratio INH-PA monohydrate cocrystal have been characterized by the terahertz time-domain (THz-TDS) and Raman spectroscopy. The THz absorption spectra displayed that there were obvious differences between the peaks of experimental cocrystal and the parent materials, and the same situation was found in Raman vibrational spectra. In addition, density functional theory (DFT) was applied to simulating and optimizing the structure of INH-PA monohydrate cocrystal and supplied corresponding vibrational modes. Our results provided a unique method to characterize the formation of INH-PA monohydrate cocrystal at the molecular-level and a lot of information about cocrystal structure and intra-molecular and/or inter-molecular hydrogen bond interactions in the emerging pharmaceutical cocrystal fields.

## 1. Introduction

**Figure 1:**
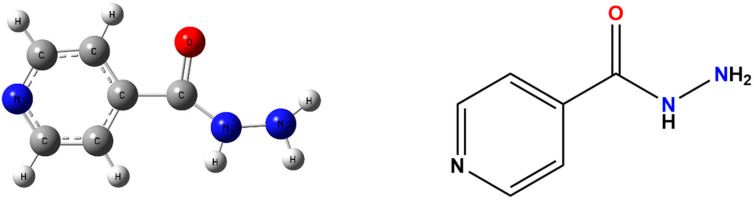
Molecular structure of INH.

Tuberculosis (TB) is a global emergency case announced by the World Health Organization (WHO) and among the top 10 causes of death [1,2]. The International Union against TB and Lung Diseases has recommended the use of fixed-dose combinations (FDCs) of anti-TB drugs to combat monotherapy and drug resistance [3,4]. Isoniazid (INH, as shown in Figure 1), the principal component in the so-called ‘triple therapy’ and different FDCs [5], tends to degrade in the FDCs involving INH and rifampicin (RIF). Moreover, oxidative stress will occur in the patients treated with anti-TB drugs such as INH and RIF, which tend to cause hepatotoxicity [6]. Therefore, in order to improve its pharmaceutical performance and enhance the stability of tablet formulations, it is urgent to explore new solid forms [7].

**Figure 2:**
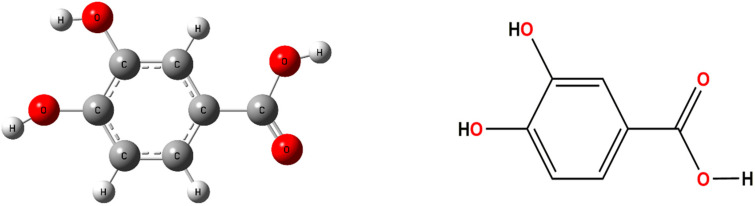
Molecular structure of PA.

Cocrystal is generally defined as the crystalline single-phase materials [8] formed by two or more solid-state neutral compounds in a certain stoichiometric ratio under the influence of inter-molecular interactions [9]. Compared with cocrystal, salt formation is limited to acid–base reaction. However, cocrystallization provides different avenues, where any pharmaceutical substance may form cocrystals without having ionizable groups [10]. Additionally, on the premise of almost no effect on the chemical structure, intrinsic biological activity and pharmacological activity of APIs [11], cocrystallization strategy can significantly optimize the physicochemical properties and clinical performance of specific APIs [12,13]. Protocatechuic acid (PA, whose molecular structure is shown in Figure 2), as a typically natural antioxidant and antibacterial agent, has been used in pharmacology and food industry because of its antioxidant activity by eliminating free radicals via donating H-atom [14]. Additionally, containing the special hydroxyl-benzoic structures, as the naturally occurring phenolic acids [15], PA possesses a great deal of hydrogen bonding sites including the carboxylic acid group and the hydroxyl groups used as both hydrogen bond donor and hydrogen bond acceptor. Therefore, PA is an ideal co-former that is prone to form steady inter-molecular hydrogen bonds with other molecules.

In the past few years, many new solid forms involving INH have been studied, especially the cocrystallization of INH and different co-forms. Pawel Grobelny et al. [16] firstly reported the cocrystal of the anti-tuberculosis drugs INH and pyrazinamide at a 1:1 molar ratio by using X-ray diffraction (XRD), differential scanning calorimetry (DSC) and thermal gravimetric analysis (TGA) techniques, which included two symmetrical independent COOH…Npyridine hydrogen bonds. Liu Fang et al. [17] synthesized and characterized the cocrystal of INH and syringic acid using the technology of XRD and DSC, presenting a new opportunity for effectively reducing the hepatotoxicity of INH. In order to solve the problem of oxidative stress and low stability in tablet formulations involving INH, Mashhadi et al. prepared and characterized the INH-antioxidant hydroxybenzoic acid [18] cocrystals and INH-PA cocrystal [19] by the technology of infra-red (IR) spectroscopy, XRD and DSC. In addition, there are certain studies on the cocrystals regarding polyphenols [20] and aromatic carboxylic acids [21] as co-formers.

Most of the studies of cocrystals relating to INH above are implemented by a few traditional ways such as differential scanning calorimetry (DSC), X-ray diffraction (XRD), which are time-consuming in some cases or not detailed enough about the structures of molecules and the influence of intra-molecular and/or inter-molecular interactions within the cocrystals [22]. Thus, the vibrational spectroscopy including Fourier transformation infrared (FTIR), Raman and the emerging terahertz (THz) spectroscopy with high sensitivity and selectivity [23,24], providing a wealth of information about the structure of molecules, intra-molecular and/or inter-molecular interactions [25], is considered to be beneficial to solid-state characterization. Especially, THz spectroscopy can provide the finger-print spectra referring to special molecular structure [26,27] and much information about the weak collective molecular vibrations and various crystalline structural changes in the low frequency region, especially below 3.0 THz [28,29].

In the present work, the monohydrate cocrystal between INH and PA was synthesized by slow solvent evaporation and THz and Raman spectral techniques were used to observe the fingerprint information from INH, PA, their physical mixture and cocrystal. The terahertz spectrum is composed of a mixture of hydrogens, weak covalent bonds, ionic bonds and van der Waals force in the solid states [30]. These complex and highly entangled molecular interactions make the terahertz spectrum results difficult to explain, so it needs relatively complex calculation and simulation [31]. Density functional theory (DFT), as a very useful means of quantum mechanics calculation, is able to explain and assign in detail the complex molecular motions obtained from THz experiments and understand the basic origin of material properties [32]. Combined with DFT calculations, terahertz spectroscopy can be used to understand the vibrational motions in ordered crystal structures [1]. Comparing the experimental results with the theoretical results, the structural changes of molecules and the interactions between and within molecules could be better comprehended in the cocrystal formation process.

## 2. Materials and Methods

### 2.1. Material Preparation and the Synthesis of Cocrystal

Anhydrous solid-state INH (purity 98%) and sample PA (purity 99%) were acquired by J&K Chemical Company (Shanghai, China). Methanol was purchased from Hangzhou Gaojing Fine Chemical Company (Hangzhou, China). All chemicals were used as received without further purification. 

The physical mixture of parent materials was obtained by mixing the two compounds at a molar ratio of 1:1 in a glass vial by using a vortex mixer. The cocrystal was obtained by slow solvent crystallization, which was commonly used in the preparation of cocrystal. INH (0.137 g, one mmol) and PA (0.154 g, one mmol) were respectively dissolved in a mixture of 20 mL methanol and de-ionized water (1:1 volume ratio; details about the generation and characterization of de-ionized water used could be found in materials of the supporting information), and then mixed together. The mixed solution was heated to 70 °C for 10 min and then held in slow evaporation for 15 days. The cocrystal was obtained by isolating the orange prism like crystals at room temperature. The separated cocrystal weighing 0.25 g was taken out and gently ground to make its average particle size several microns, so as to minimize the scattering effect of sample particles in terahertz spectrum measurement. Then the cocrystal substance was put into the tablet press to impose 4 MPa pressure for about two minutes, and the physical mixture performed the same operation. The sample prepared according to the above steps was a circular plate with a diameter of 13 mm and a thickness of 2 mm, which was put into a sealed bag for subsequent THz detection. For the detection of Raman spectrum, only a small amount of powder sample was needed without further sample preparation.

### 2.2. THz/Raman Device and Detection

THz spectra of INH, PA, their physical mixture and cocrystal were detected by THz-TDS Z2 system (Zomega Co., New York, NY, USA), which was applied to investigate the THz transmission spectra of target substances based on photoconductive switches. The optical pulse sequence was from Ti: Sapphire oscillator ultrafast laser pulse system (Spectra Physics, Owen, CA, USA) with the frequency 75 MHz, the pulse width 100 fs and the center wavelength 780 nm. Due to the strong absorption of THz radiation by water, nitrogen gas with high purity was continuously flushed into the detector during the experiment. Three measurements for each set of sample and reference were carried out and took the average value as the ultimate spectral data. Since the outputs of the experiment were time domain spectra of the electric field intensity of THz, they need to be converted into corresponding frequency domain spectra by fast Fourier transform (FFT) process, and the THz absorption spectra were acquired by dividing the sample frequency response by that of the reference. The Raman spectra of the above substances were detected by the Fourier transform Raman (FT-Raman) spectrometer (Spectra Physics, Owen, CA, USA) after over 500 scans at 2 cm^−1^ spectral resolution over the wavenumber range between 150 and 3500 cm^−1^. A diode pumped (wavelength at 1064 nm) solid-state laser was selected as the near-infrared light source with 150 mW operating power and the total analysis time for each sample was of the order of 5 min intervals.

### 2.3. DFT Theoretical Calculations

**Figure 3:**
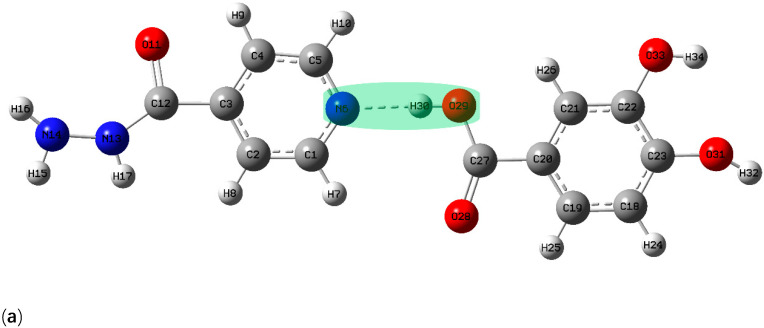

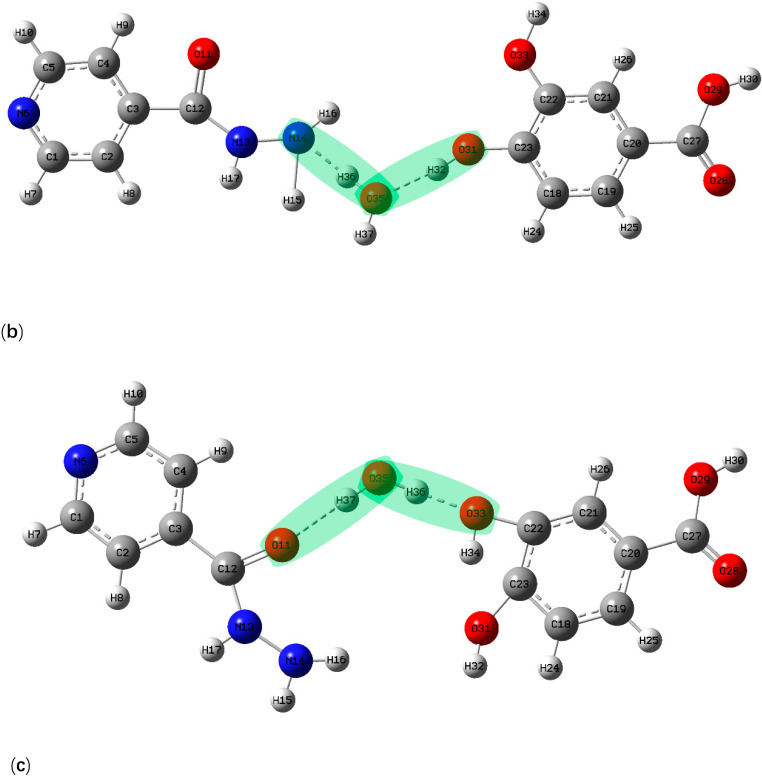
Schematic diagram of molecular structure for possible theoretical cocrystal (**a**) form I, (**b**) form II, (**c**) form III.

The crystal engineering method of supramolecular heterosynthesis between the API and the coformer is an effective strategy for the rational selection of suitable coformers and the design of new pharmaceutical cocrystals. It is important to understand the hydrogen bond interactions, which lead to strong heterosynthons in the cocrystal compared to homosynthons in individual components [33]. Therefore, it is necessary to theoretically predict and analyze the terahertz spectra of crystal materials. On the one hand, the less accurate semi-empirical method that allows full crystal calculations of the terahertz vibrations, not limited to one construction unit can reduce the calculation time [34]. In addition, when the correction factors are used to compensate the systematic deviations, ab initio and DFT methods are considered to be very accurate in predicting molecular vibrations. In this paper, Gaussian 09 software was used to simulate and optimize the theoretical structures of INH, PA and their possible cocrystal forms, and B3LYP was selected as a credible DFT geometry majorization method in the computational process [35,36]. Based on the structural characteristics of INH and PA, three possible cocrystal forms were simulated. In form I, the hydrogen bond was formed by pyridine N–carboxylic acid synthons of two raw materials (as shown in Figure 3a). In form II and form III, hydrogen bonds were formed between raw materials and single water molecule, composing different ring structures (as shown in Figure 3b,c). Although Mashhadi et al. [19] have synthesized and characterized the cocrystal of INH and PA by means of XRD and DSC, there is no structural report on the analysis of the cocrystal of the above substances based on THz and Raman spectra. To further characterize the hydrogen bond motifs shown within the INH-PA hydrate cocrystal structure, the results of THz and Raman spectra were combined with that of DFT simulation, which is of great significance to better comprehend the structural characteristics and vibrational modes of INH-PA hydrate cocrystal. In the simulated THz and Raman spectra, a full-width half-maximum (FWHM) value of 4.0 cm^−1^ was used to convolute the Lorentzian line shapes into the calculated vibrational modes.

## 3. Results and Discussion

### 3.1. THz Spectral Characterization and Analysis

**Figure 4:**
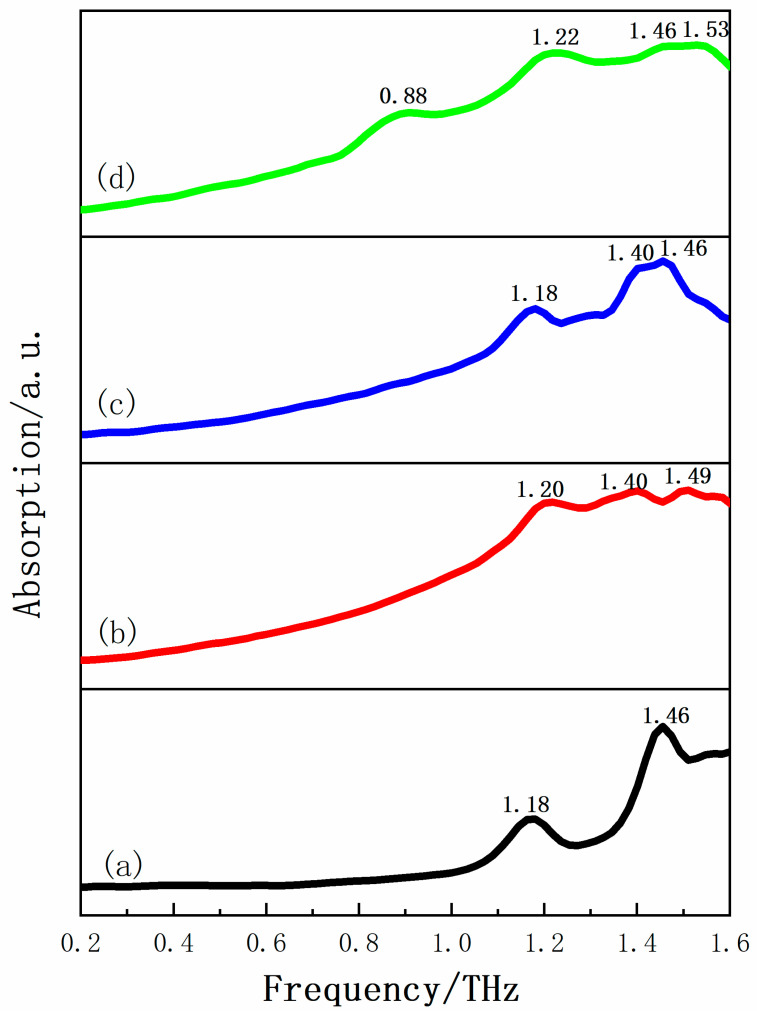
The THz spectra of (**a**) INH, (**b**) PA, (**c**) physical mixture and (**d**) INH-PA monohydrate cocrystal in 0.2–1.6 THz spectral region.

The low frequency vibrational spectra of molecules provide many useful values for molecular vibration, rotation modes, and some weak inter-molecular interactions such as hydrogen bond interactions, van der Waals forces and so on. THz spectroscopy can be used to obtain such low frequency vibrational information of crystalline compounds, which makes it a vigorous analytical tool for studying the structure and inter-molecular interaction of various molecules [37]. The THz absorption spectrum of INH, PA, their physical mixture and corresponding INH-PA monohydrate cocrystal in the range of 0.2–1.6 THz is shown in Figure 4. It could be clearly seen that INH has two distinct characteristic peaks at 1.18 and 1.46 THz, which makes it possess good spectral resolution. Additionally, PA engendered three relatively flat absorption peaks at 1.20, 1.40 and 1.49 THz. In addition, the physical mixture mainly has three obvious absorption peaks, which are located at 1.18, 1.40 and 1.46 THz, respectively. There is no new characteristic peak, but a simple superposition of respective absorption spectra of the two original substances in the THz absorption spectrum of the physical mixture. Nevertheless, the THz absorption spectrum of INH-PA monohydrate cocrystal shows a few characteristic peaks at 0.88, 1.22, 1.46 and 1.53 THz differing from both two individual raw substances and their simple physical mixture. Therefore, above-mentioned characteristic peaks indicate that INH and PA have successfully synthesized new substance by slow evaporation under the influence of inter-molecular and/or intra-molecular interactions. This also verified the ability of THz spectroscopy to supply obvious fingerprint information for the molecular structure of solid-state crystal. Moreover, the cocrystal was characterized by the H-NMR spectrum as shown in Figure S1, which proved that the compound did contain water but did not contain other solvents.

**Figure 5:**
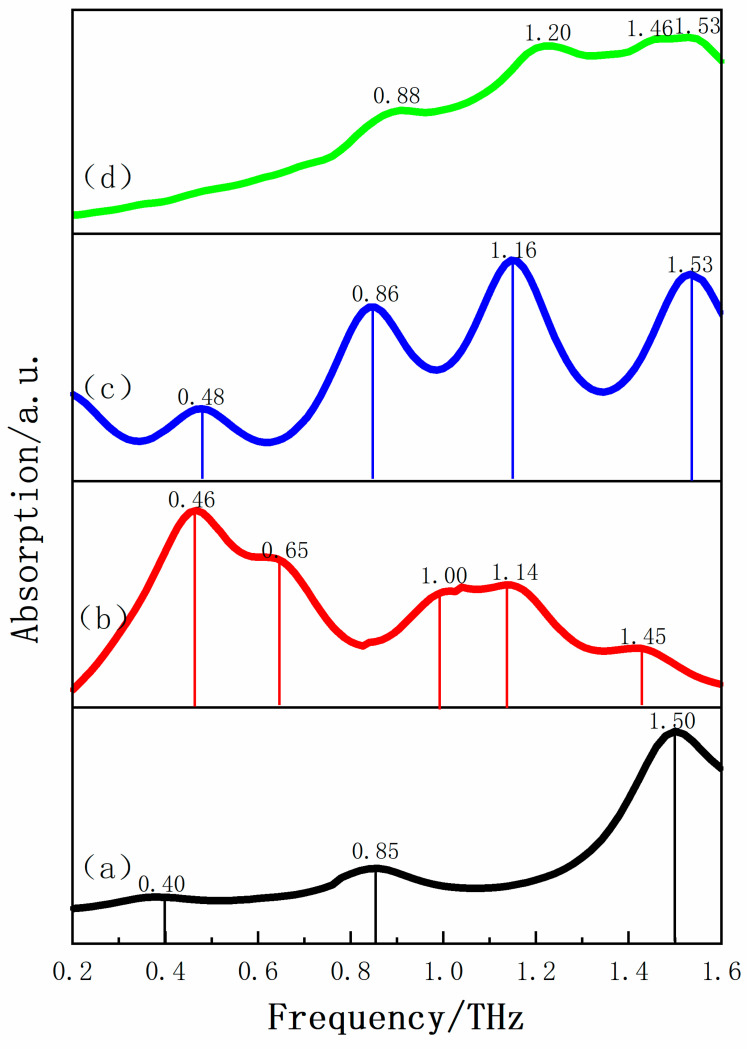
Comparisons of THz absorption spectra of (**a**) theoretical cocrystal form I, (**b**) theoretical cocrystal form II, (**c**) theoretical form cocrystal III and (**d**) experiment result.

Three possible theoretical cocrystal forms were simulated in this paper. In form I, the hydrogen bond was formed by pyridine N–carboxylic acid synthons of two raw materials (as shown in Figure 3a). In form II and form III, hydrogen bonds were formed between raw materials and single water molecule (as shown in Figure 3b,c). The comparisons of THz absorption spectra between experimental and theoretical results are shown in Figure 5. There are some obvious characteristic peaks at 0.4, 0.85 and 1.50 THz for theoretical form I and 0.46, 0.65, 1.10, 1.14 and 1.45 THz for theoretical form II, respectively. Similarly, theoretical form III reveals characteristic peaks with high peak intensity and resolution at 0.48, 0.86, 1.16 and 1.53 THz, respectively. However, the theoretical form I lacks the characteristic peak at 1.20 THz and the theoretical form II does not appear the corresponding characteristic peak at 0.88 THz, compared with the experimental absorption spectra. Surprisingly, the absorption peaks of theoretical form III at 0.86, 1.16 and 1.53 THz are in good agreement with the experimental characteristic peaks at 0.88, 1.20 and 1.53 THz. Therefore, the THz spectra of the theoretical cocrystal form III is more consistent with that of experimental results, which also proves the formation of cocrystal. Although, the absorption peak of theoretical cocrystal form III is unable to match the experiment at 0.48 and 1.46 THz and a certain red shift exists at 0.86 and 1.16 THz, which may result from the fact that the theoretical calculation is carried out at absolute zero temperature while the actual THz spectral detection is measured at room temperature [38]. Additionally, the theoretical simulation only calculates one construction unit of the whole cocrystal molecular network, while the experimental cocrystal spectrum is used to characterize the whole solid-state cocrystal molecular network. In addition, different computing settings will have a certain impact on consistency between theory and experiment as well.

**Figure 6:**
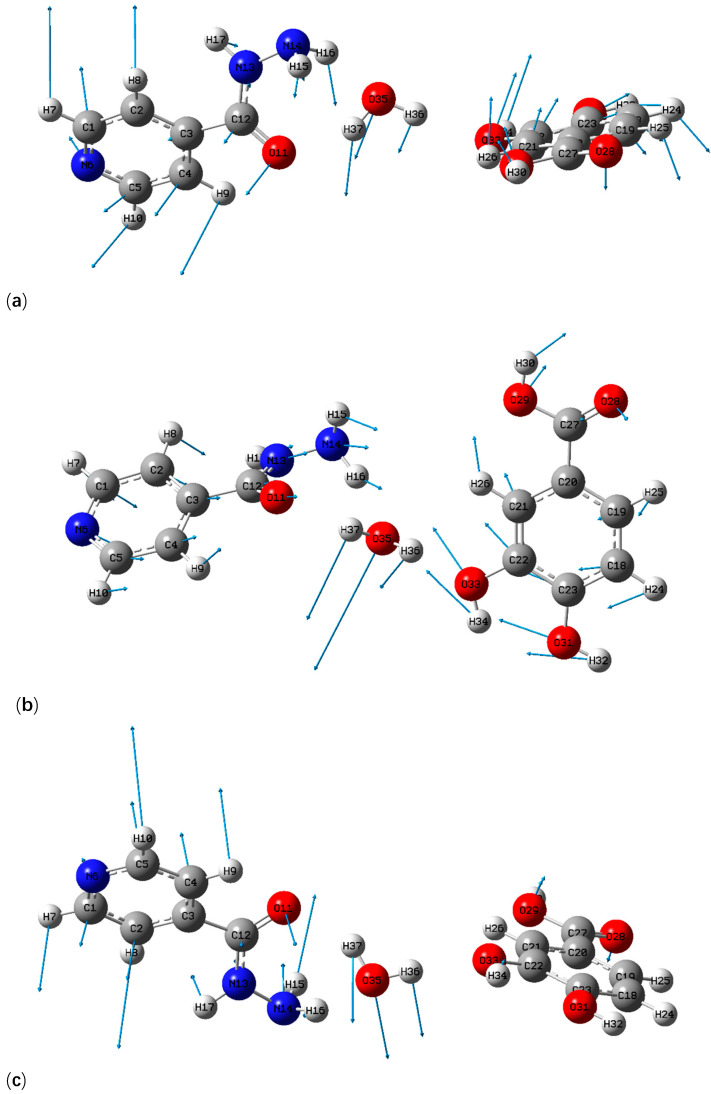
Vibrational mode description of INH-PA monohydrate cocrystal at positions of (**a**) 0.88, (**b**) 1.20 and (**c**) 1.53 THz.

GaussView is one kind of software specially designed for Gaussian, whose main purpose is to construct Gaussian input file and to display Gaussian output calculation results in the form of a graph. Through the GaussView software, the various vibrational modes of theoretical cocrystal form III between INH and PA (shown in Figure 6) could be conveniently observed and the vibrational modes at different characteristic peaks are assigned, as shown in Table 1. Corresponding to the absorption peak of experimental cocrystal at 0.88 THz, the characteristic peak of theoretical cocrystal form III at 0.86 THz is mainly caused by the out of plane rocking vibrations of INH and PA molecules. The out of plane rocking vibration of INH and in-plane rocking vibration of PA make the theoretical cocrystal form III format an obvious characteristic peak at 1.16 THz, which is consistent with the absorption peak of the experimental cocrystal at 1.20 THz. The crystal vibration of the absorption peaks at 1.53 THz of the experimental and theoretical cocrystal is mainly owing to the out of plane rocking vibration from the ring of INH and the torsional vibrations from O11-C12-N13-H17 of INH and H30-O29-C27-O28 of PA. Moreover, the above three vibrational modes are accompanied by the vibration of water molecules (H36-O35-H37), which is consistent with the theoretical simulation of INH, PA and water molecule forming O35-H36…O33, O35-H37…O11 and O33-H34…N14 inter-molecular hydrogen bonds. Through the analysis of vibrational modes, it can be proved that the INH-PA monohydrate cocrystal is formed chiefly due to the generation of inter-molecular hydrogen bonds, which plays a key role in changing the molecular structure and makes the vibrational modes different from those of the original parent materials. Therefore, THz spectroscopy can be applied to characterize the hydrogen bond effect sensitively, owing to its absorption mechanism affected by intra-molecular and inter-molecular vibrational excitation [39]. In addition, the powder diffraction experiments of INH, PA and their hydrate cocrystal were performed, and the powder diffraction pattern of INH-PA cocrystal was simulated according to the crystal structure data from the Cambridge Crystallographic Data Centre (CCDC). The experimental X-ray diffraction (XRD) pattern is almost consistent with the calculated pattern, which further confirms the formation of INH-PA monohydrate cocrystal (PXRD pattern is displayed as shown in Figure S2 in Supporting Information).

### 3.2. Raman Spectral Characterization and Analysis

**Figure 7:**
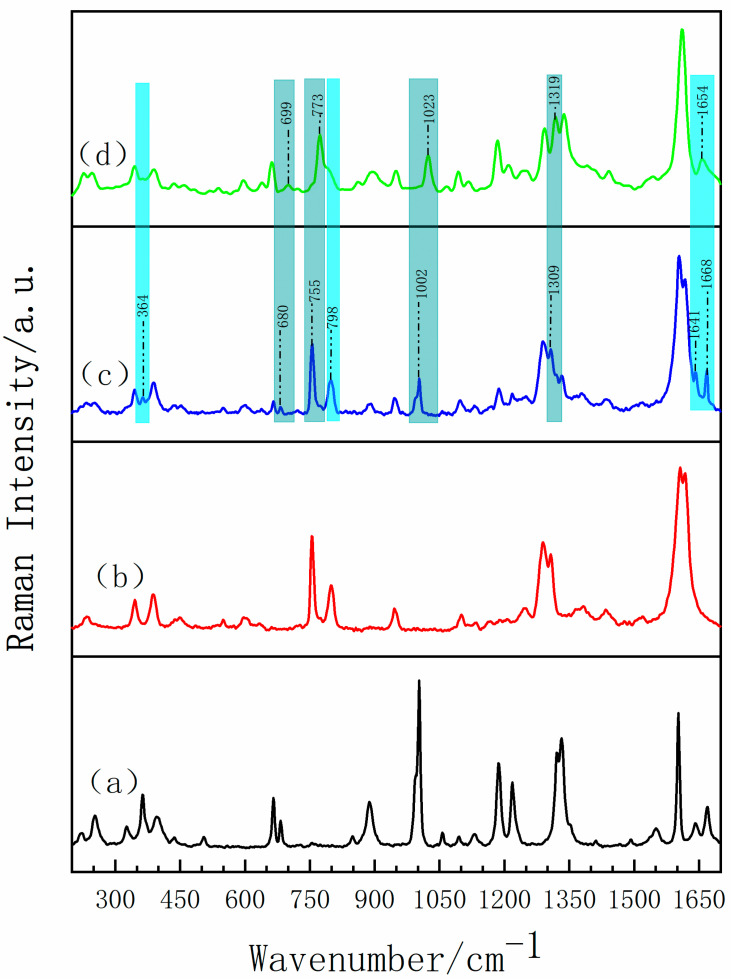
Experimental Raman spectra of (**a**) INH, (**b**) PA, (**c**) physical mixture and (**d**) INH-PA monohydrate cocrystal.

Raman spectroscopy has become one of the preferred methods to study the solid-state polymorphism of drugs (polycrystalline, solvent, hydrate, salt, eutectic, etc.), not only because of its sensitivity to structural changes, but also its rapidity, non-destruction, no sample preparation and capability of characterization in the water environment [40]. Different from THz spectroscopy based on absorption mechanism, Raman spectroscopy mainly relies on light scattering to obtain an abundance of structure information from target substances, such as the vibrational transitions in molecules, electronic polarizabilities, and vibrational energy transitions of the molecules [41,42]. Figure 7 shows the Raman vibrational spectra of INH, PA, their physical mixture and INH-PA monohydrate cocrystal acquired by experiments. As it can be seen, the characteristic peaks of the physical mixture are mainly attributed to the simple linear superposition of the two raw materials, so it does not involve intra-molecular and/or inter-molecular interactions such as hydrogen bonding just during the simple mixing process, which is in good agreement with the above THz spectral results. Compared with the Raman spectra of INH-PA physical mixture, some corresponding characteristic peaks were not discovered at 364 and 798 cm^−1^ in the Raman spectra of INH-PA monohydrate cocrystal. Additionally, there is a new characteristic peak at 1654 cm^−1^ in Raman spectra of INH-PA monohydrate cocrystal, which are not found in the original parent materials and physical mixture. For the convenience of observation, the above-mentioned two kinds of characteristic peaks are covered with cyan shadows in Figure 7. In addition, corresponding to the characteristic peaks of physical mixture at 680, 755, 1002 and 1309 cm^−1^, the relevant characteristic peaks of cocrystal have a certain blue shift and/or red shift, respectively, located at 699, 773, 1023 and 1319 cm^−1^. Such characteristic peaks are shadowed with light green color in Figure 7. According to the analysis of the above spectral diversity, it can be inferred that a new substance between INH and PA is produced, and the characteristic peaks of the substance which reflect the molecular structure are completely different from those of the original materials in some specific locations.

**Figure 8:**
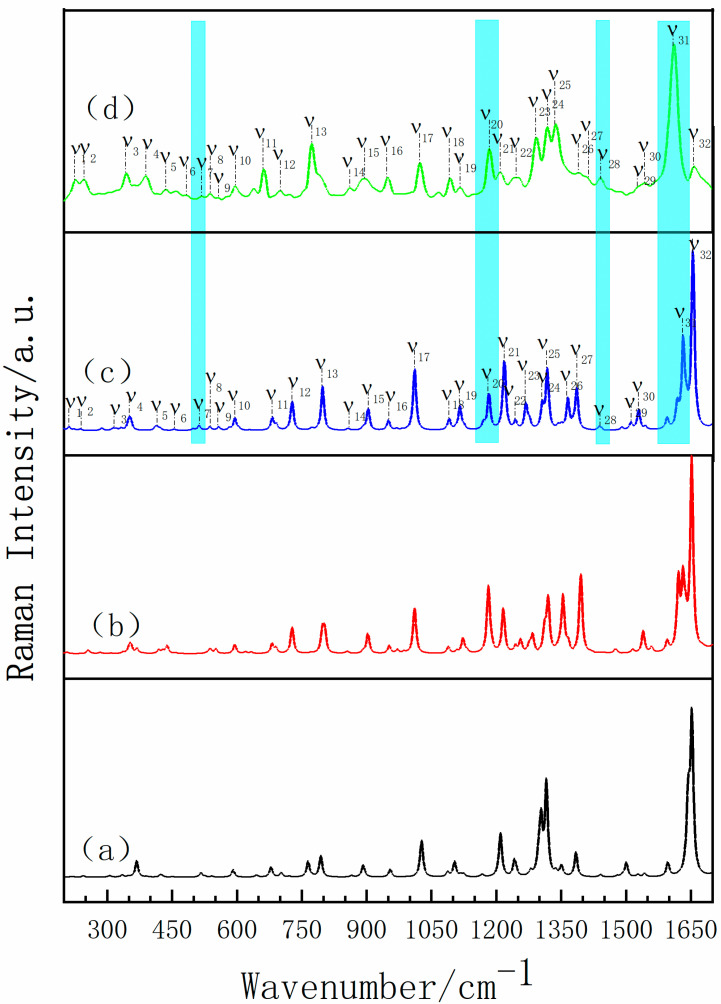
Comparisons of Raman spectra of (**a**) theoretical cocrystal form I, (**b**) theoretical cocrystal form II, (**c**) theoretical form cocrystal III and (**d**) experiment result.

The Raman spectra of the experimental results compared with the theoretical calculation are shown in Figure 8 (the theoretical cocrystal consists of three possible forms) and some differences among the three theoretical spectra are observed. Corresponding to the characteristic peak at 1184 cm^−1^ in the experimental Raman spectrum, theoretical form II and theoretical form III are in good agreement, while theoretical form I lacks the relevant characteristic peak. Comparing the theoretical form II with the theoretical form III, the characteristic peak of the theoretical form III at 1442 cm^−1^ is well matched with that of experiment, but the theoretical form II is short of such characteristic peak. Additionally, located in the high frequency region of 1610 cm^−1^, the number of characteristic peaks of form III is more consistent with the experimental spectrum. Therefore, the Raman spectrum from theoretical form III is in better agreement with the experimental results, which is consistent with the above THz spectrum results and proves the formation of cocrystal. In addition, the distinct vibrational modes of INH-PA monohydrate cocrystal are assigned in Table 2. Combined with the theoretical simulation results, it can be obtained that the characteristic peak at 798 cm^−1^ is mainly attributed to the scissoring vibration of O33-C22-C21-H26, deformation vibration of PA ring and the out of plane rocking vibration of H36-O35. Another characteristic peak with high resolution at 1318 cm^−1^ is mainly formed by the in-plane rocking vibration of H17-N13-C12, the out of plane rocking vibration of H15-N14-H16 and stretching vibration of N14-N13. In addition, two obvious characteristic peaks located in the high frequency region are accompanied by the vibration of the water molecule during the crystal vibration. For instance, the characteristic peak at 1632 cm^−1^ can be considered as the result of deformation vibration of INH ring and the scissoring vibration of water (H36-O35-H37). Additionally, the characteristic peak at 1655 cm^−1^ can be attributed to the deformation vibration of PA ring and the scissoring vibration of H34-O33-C22 and water (H36-O35-H37). Among them, O35-H36... O33 and O35-H37... O11 are the two important hydrogen bonds forming the cocrystal structure. By analyzing the vibrational modes of the four Raman characteristic peaks with high resolution, it can be seen that most of the vibrations of INH-PA hydrate cocrystal are influenced by inter-molecular interactions, especially the effect of inter-molecular hydrogen bonds between INH, PA and water molecule. Therefore, the hydrogen bonds play an important role in the formation of INH-PA monohydrate cocrystal.

**Figure 9:**
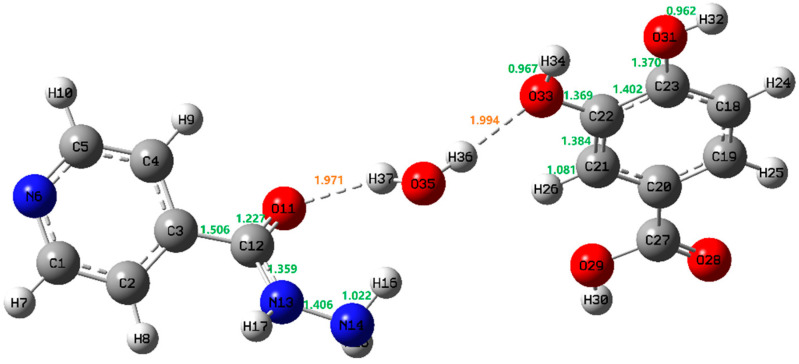
Typical bond length/Å of INH-PA monohydrate cocrystal.

It is well known that the change of bond length between different atoms is the most direct reason for the characteristic vibration mode information exhibited in the Raman spectra of various compounds [41,42]. Therefore, to further understand the specific micro-molecular structure of INH and PA monohydrate cocrystal, the research on the typical bond length change of cocrystal structure and the relationship with corresponding characteristic peaks were also added. Typical bond lengths of INH-PA monohydrate cocrystal are shown in Figure 9 and some typical bond length variations upon the formation of cocrystal are also given in Table 3. The bond lengths of O11-C12, N13-N14, N14-H15 and N14-H16 from INH changed by 0.251, 0.071, 0.016 and 0.022 Å, respectively, while H34-O33 and O33-C22 from PA changed by 0.007 and 0.061 Å, respectively. Moreover, the bond lengths of H37-O35 and O35-H36 in water molecule also increased. The change of the length of above-mentioned chemical bond is closely related to the two inter-molecular hydrogen bonds (O35-H37…O11 and O35-H36…O33) forming INH-PA monohydrate cocrystal. In addition, it can be seen from Table 3 that the chemical bonds with the largest bond length change (including O11-C12 of INH and O33-C22 of PA) form inter-molecular hydrogen bonds with water molecule, which greatly contribute to the vibrational modes at 798, 1318, 1632 and 1655 cm^−1^ in the above Raman spectra, which also confirms the important influence of inter-molecular hydrogen bonding on the formation of cocrystal. Combining the results of chemical bond length variations with THz and Raman spectra, it can help us better understand the offset, emergence and vanishing of characteristic peaks in the spectrum of INH-PA monohydrate cocrystal and provides us with abundant information about the molecular structure and inter-molecular interactions (particularly the hydrogen bonds between raw substances).

## 4. Conclusions

In this paper, the vibrational spectra of solid-state INH, PA, physical mixtures and INH-PA monohydrate cocrystal were obtained by THz and Raman spectra. It can be perceived that there are some differences between the vibrational spectra of INH-PA monohydrate cocrystal and those of original materials. Three possible theoretical cocrystal forms were simulated by DFT so as to compare with the experimental spectra, then the vibrational modes at different absorption peaks were assigned according to the above simulation results. According to the above analysis results, it can be concluded that the monohydrate cocrystal between INH and PA is formed on the score of non-covalent bond interactions between molecules, particularly the influence from inter-molecular hydrogen bonds. Through the comparison of three theoretical cocrystal forms, the theoretical cocrystal form III (INH, PA and water form O35-H37…O11 and O35-H36…O33 inter-molecular hydrogen bonding) is more consistent with the experimental results. In addition, the study on typical hydrogen bond length also confirmed the important role of hydrogen bonds in the formation of cocrystal. Raman and terahertz spectroscopic techniques are complementary to the investigation into vibrational modes and micro-molecular structure of INH-PA monohydrate cocrystal. The study shows the superiority of vibrational spectroscopy adequately certifying the molecular structure and inter-molecular hydrogen bond interactions in the study of specific cocrystals, which provides theoretical and experimental basis for the structural research of emerging pharmaceutical cocrystal fields at the micro-molecular level. 

## Figures and Tables

**Table 1:** Vibrational mode assignment of the monohydrate cocrystal formed between INH and PA shown in THz spectra.

Experimental Results f/THz	Theoretical Results f/THz	Mode Assignment
0.88	0.86	Out of plane rocking vibration of INH and PA
1.20	1.16	Out of plane rocking vibration of INH and in-plane rocking vibration of PA
1.46	-	
1.53	1.53	Out of plane rocking vibration of the ring of INH, torsional vibration of O11-C12-N13-H17 and H30-O29-C27-O28

**Table 2:** Vibrational mode assignment for characteristic peaks of the monohydrate cocrystal formed between INH and PA shown in Raman spectra.

Mode	Theoretical Wavenumber/cm^−1^	Experimental Wavenumber/cm^−1^	Mode Assignment
ν_1_	212	225 (w)	ω(H32-O31-C23-C22-O33-H34), Def(R2)
ν_2_	238	244 (w)	τ(H32-O31-C23-C18-H24, O33-C22-C21-H26)
ν_3_	316	343 (w)	δ(H34-O33-C22-C23-O31-H32), ω(H36-O35-H37)
ν_4_	354	387 (w)	ω(H16-N14-H15), ρ(H36-O35-H37), δ(C2-C3-C4)
ν_5_	416	433 (vw)	ω(H34-O33-C22), ρ(H36-O35-H37, H15- N14-H16)
ν_6_	456	483 (vw)	Def(R2), ρ(H36-O35-H37)
ν_7_	513	516 (vw)	ω(H17-N13-C12), ρ(H36-O35-H37, H15-N14-H16)
ν_8_	538	537 (w)	τ(H38-O29-C27, H25- C19-C18-H24)
ν_9_	558	557 (vw)	ω(H36-O35-H37, H34-O33-C22)
ν_10_	595	595 (w)	Def(R2), τ(36-O35-H37)
ν_11_	682	663 (m)	Def(R1)
ν_12_	728	700 (vw)	δ(H30-O29-C27-O28), Def(R2)
ν_13_	798	775 (s)	δ(O33-C22-C21-H26), Def(R2), ω(H36-O35)
ν_14_	857	859 (vw)	ω(H7-C1-C2-H8, H9-C4-C5-H10)
ν_15_	903	895 (m)	δ(O11-C12-N13), ω(H15-N14-H16), ρ(H36-O35-H37)
ν_16_	950	950 (m)	δ(C19-C20-C21, H24-C18-C19-H25)
ν_17_	1012	1023 (m)	Def(R1), ω(H15-N14-H16)
ν_18_	1092	1093 (w)	ρ(H26-C21-C20), Def(R2)
ν_19_	1116	1115 (w)	δ(H25-C19-C18-H24, H32-O31-C23-C18-H24)
ν_20_	1183	1184 (m)	ρ(H30-O29-C27), δ(H25-C19-C18-H24, H26-C21-C20-C27)
ν_21_	1219	1213 (m)	ρ(H8-C2-C3), ω(H15-N14-H16)
ν_22_	1244	1245 (w)	δ(H7-C1-C2-H8, H10-C4-C5-H9), ω(H15-N14-H16)
ν_23_	1270	1292 (w)	ρ(H26-C21-C22, H25-C19-C18-H24)
ν_24_	1307	1319 (m)	ρ(H24-C18-C19-H25), Def(R2)
ν_25_	1318	1336 (s)	ρ(H17-N13-C12), ω(H15-N14-H16), θ(N14-N13)
ν_26_	1366	1390 (vw)	δ(H30-O29-C27), ρ(H25-C19-C18-H24)
ν_27_	1386	1409 (vw)	δ(H34-O33-C22, H32-O31-C23)
ν_28_	1440	1440 (vw)	δ(H7-C1-C2-H8, H10-C4-C5-H9)
ν_29_	1512	1524 (vw)	ρ(H17-N13-C12, H7-C1-C2-H8, H10-C4-C5-H9)
ν_30_ ν_31_ ν_32_	1530 1632 1655	1542 (vw) 1610 (s) 1656 (m)	δ(H17-N13-C12), ρ(H7-C1-C2-H8, H10-C4-C5-H9) Def(R1), δ(H36-O35-H37) Def(R2), δ(H34-O33-C22, H36-O35-H37)

Vw—very weak; w—weak; m—medium; s—strong, θ—stretching; ρ—in-plane rocking vibration; δ—scissor; ω—out of plane rocking vibration; τ—torsion; Def—deformation.

**Table 3:** Changes of typical chemical bond lengths between INH, PA and the INH-PA monohydrate cocrystal.

Chemical Bond	Bond Length/Å		
	INH	PA	Cocrystal
O11-C12 C12-C3	1.478 1.588	-	1.227 1.506
C12-N13	1.470	-	1.359
N13-N14	1.477	-	1.406
N14-H15	1.000	-	1.016
N14-H16	1.000	-	1.022
H34-O33		0.96	0.967
O33-C22	-	1.43	1.369
C22-C21 C21-H26	-	1.394 1.100	1.384 1.081
C22-C23	-	1.395	1.402
C23-O31	-	1.430	1.370
O31-H32	-	0.960	0.962

## Data Availability

Not applicable.

## Supplementary Materials

The following are available online at https://www.mdpi.com/article/10.3390/pharmaceutics13081303/s1, Figure S1: H-NMR spectrum of INH-PA monohydrate cocrystal in the region of 0.5–8.5 ppm. The signal at 2.51 ppm is DMSO from the solvent., Figure S2: PXRD comparison of INH (a), PA (b), INH-PA monohydrate cocrystal from experiment (c) and INH-PA monohydrate cocrystal from theoretical simulation (d) in the 2θ range 5–50°, Figure S3: The THz spectra of INH (a), PA (b), their physical mixture (c) and INH-PA monohydrate cocrystal (d) in 0.6–1.6 THz spectral region. Experimental Details and Characterization Techniques.
