# Supplementary material for: Terahertz and Raman Spectroscopic Investigation of Monohydrate Cocrystal of Antitubercular Isoniazid with Protocatechuic Acid
Source: Pharmaceutics. 2021 Aug 20;13(8):1303. doi: 10.3390/pharmaceutics13081303 (PMC8400006; doi:10.3390/pharmaceutics13081303)
Supplement: Supplementary file 1 [file pharmaceutics-13-01303-s001.zip › pharmaceutics-1310189-supplementary.pdf]

pharmaceutics

Supplementary Materials: Terahertz and Raman Spectroscopic Investigation of Monohydrate Cocrystal of Antitubercular Isoniazid with Protocatechuic Acid

Yanhua Bo, Jiyuan Fang, Ziming Zhang, Jiadan Xue, Jianjun Liu, Zhi Hong and Yong Du

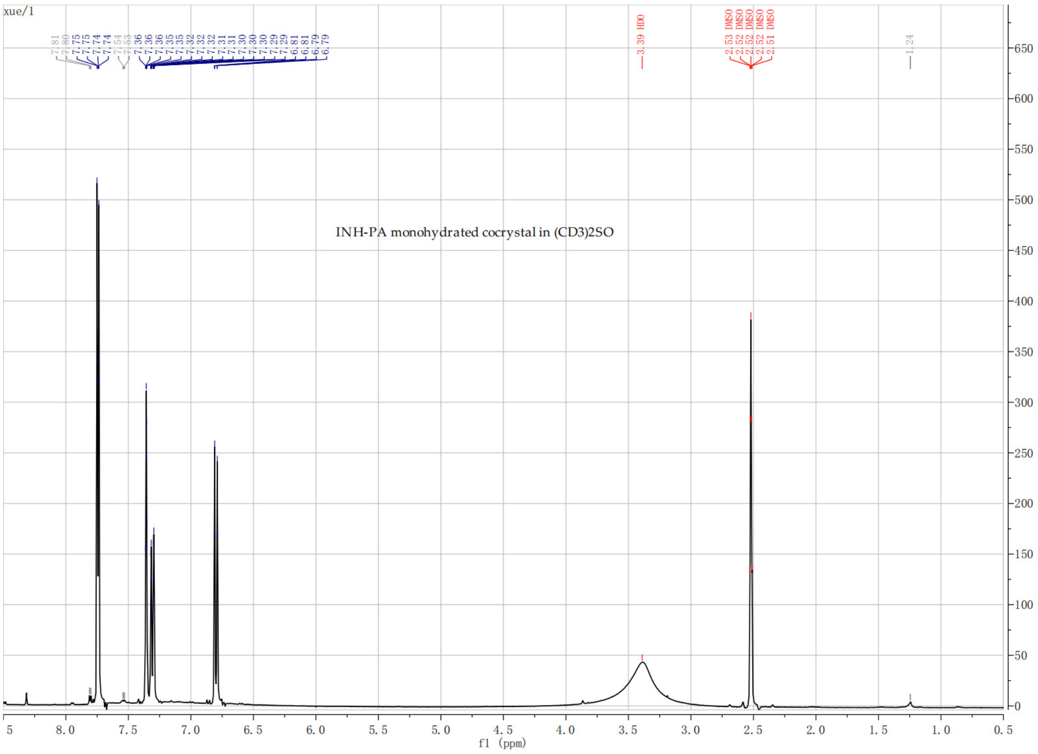

Figure S1. H-NMR spectrum of INH-PA monohydrate cocrystal in the region of 0.5–8.5 ppm. The signal at 2.51 ppm is DMSO from the solvent.

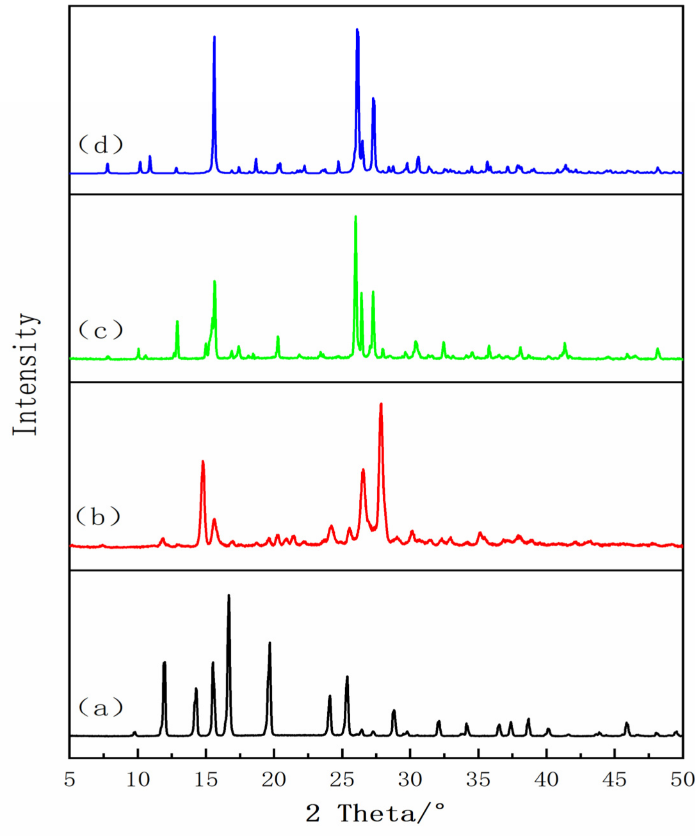

Figure S2. PXRD comparison of INH (a), PA (b), INH-PA monohydrate cocrystal from experiment (c) and INH-PA monohydrate cocrystal from theoretical simulation (d) in the 2θ range 5–50°.

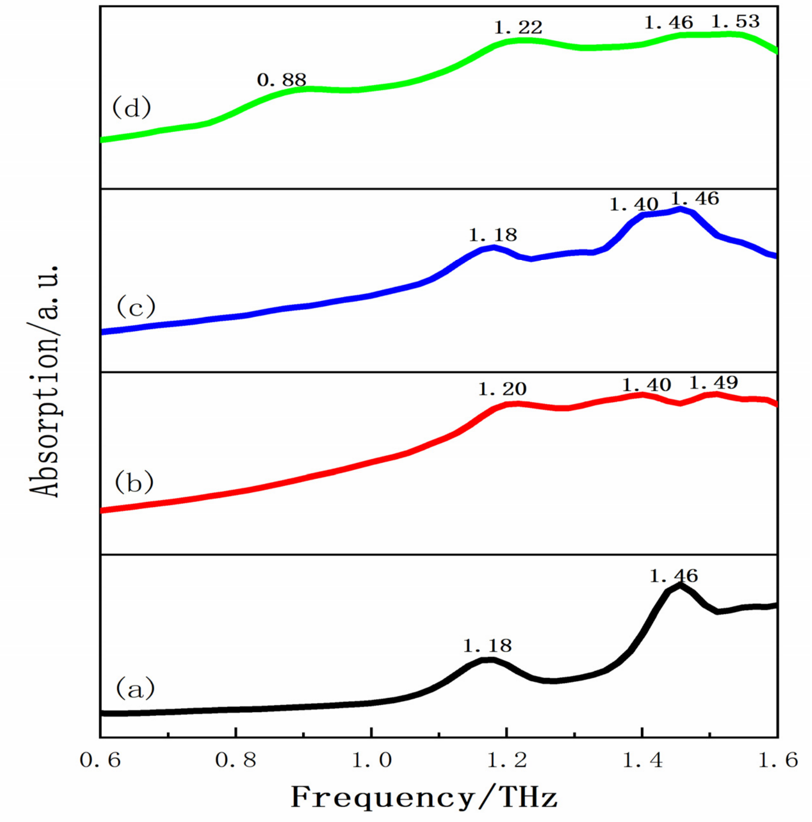

Figure S3. The THz spectra of INH (a), PA (b), their physical mixture (c) and INH-PA monohydrate cocrystal (d) in 0.6–1.6 THz spectral region.

Experimental Details and Characterization Techniques

Powder X-Ray Diffraction (PXRD) Measurements.

PXRD patterns of INH, PA, corresponding physical mixture and INH-PA monohydrate cocrystal samples were obtained by using a DX-2700 diffractometer (Dandong Haoyuan Instrument Co. Ltd. China with Cu Kα radiation) at 40 kV and 40 mA. Samples were measured in the 2θ range of 5-50° with the scan speed at 1.5°/min.

Generation of Deionized Water.

The deionized (DI) water was generated by a water purifier (OPTION-S15BP, ELGA LabWater Global Operations) using an ion-exchange column to remove the ions in water. The resistivity of DI water was around 15 MΩ·cm.

PH Measurement of Deionized Water.

PHSJ-4A pH meter (Shanghai Precision Scientific Instrument Co., Ltd.) was used to measure the pH value of DI water. The result showed that the pH value of DI water at room temperature was 6.86, which is in neutral state.

H-Nuclear Magnetic Resonance (H-NMR) Measurement.

About 10 mg of the INH-PA monohydrate cocrystal sample was dissolved in 0.6 mL of DMSO-d6 solvent and was immediately analyzed by NMR spectroscopy. The NMR measurement was performed on a BRUKER Avance AV400 MHz NMR spectrometer (Bruker, Rheinstetten, Germany) with a z-gradient unit at 300 K with 400 MHz for ^{1}HNMR spectrum employing the manufacturer's pulse programs.
